# Characterization of rumen microbiome and metabolome from oro-esophageal tubing and rumen cannula in Holstein dairy cows

**DOI:** 10.1038/s41598-023-33067-5

**Published:** 2023-04-11

**Authors:** Lais L. da Cunha, Hugo F. Monteiro, Caio C. Figueiredo, Igor F. Canisso, Rodrigo C. Bicalho, Felipe C. Cardoso, Bart C. Weimer, Fabio S. Lima

**Affiliations:** 1 Department of Forage Plants and Agrometeorology, Faculty of Agronomy, Federal University of Rio Grande do Sul (UFRGS), Porto Alegre, Brazil; 2 Department of Population Health and Reproduction, School of Veterinary Medicine, University of California, Davis, CA 95616 USA; 3 Department of Large Animal Clinical Sciences, D. H. Barron Reproductive, and Perinatal Biology Research Program, University of Florida, Gainesville, 32610 USA; 4 Department of Veterinary Clinical Sciences, Washington State University, Pullman, 99164 USA; 5 Department of Veterinary Clinical Medicine, University of Illinois, Urbana, IL USA; 6 Department of Population Medicine and Diagnostic Sciences, Cornell University, Ithaca, NY USA; 7 Department of Animal Sciences, University of Illinois, Urbana, IL USA

**Keywords:** Computational biology and bioinformatics, Microbial ecology, Applied microbiology, Bacteria, Microbial communities

## Abstract

Less invasive rumen sampling methods, such as oro-esophageal tubing, became widely popular for exploring the rumen microbiome and metabolome. However, it remains unclear if such methods represent well the rumen contents from the rumen cannula technique. Herein, we characterized the microbiome and metabolome in the rumen content collected by an oro-esophageal tube and by rumen cannula in ten multiparous lactating Holstein cows. The 16S rRNA gene was amplified and sequenced using the Illumina MiSeq platform. Untargeted metabolome was characterized using gas chromatography of a time-of-flight mass spectrometer. *Bacteroidetes*, *Firmicutes*, and *Proteobacteria* were the top three most abundant phyla representing ~ 90% of all samples. Although the pH of oro-esophageal samples was greater than rumen cannula, we found no difference in alpha and beta-diversity among their microbiomes. The overall metabolome of oro-esophageal samples was slightly different from rumen cannula samples yet more closely related to the rumen cannula content as a whole, including its fluid and particulate fractions. Enrichment pathway analysis revealed a few differences between sampling methods, such as when evaluating unsaturated fatty acid pathways in the rumen. The results of the current study suggest that oro-esophageal sampling can be a proxy to screen the 16S rRNA rumen microbiome compared to the rumen cannula technique. The variation introduced by the 16S rRNA methodology may be mitigated by oro-esophageal sampling and the possibility of increasing experimental units for a more consistent representation of the overall microbial population. Studies should consider an under or over-representation of metabolites and specific metabolic pathways depending on the sampling method.

## Introduction

The ruminant digestive tract comprises four-chambered stomachs degrading and processing the diet ingested by animals^1^. This process depends on predominantly anaerobic microorganisms inside the rumen responsible for breaking down a variety of feed particles into digestible nutrients, such as ß-linked carbohydrates into digestible sugars^2^. Furthermore, fermentation of these nutrients by ruminal microorganisms is advantageous to their growth and proliferation and provides significant precursors for the host's metabolic pathways^3^. The diet degradation process through fermentation by microorganisms starts in the particulate fraction of the rumen content. Microorganisms such as *Fibrobacter* and *Ruminococcus* adhere to fibrous polysaccharides and hydrolyze them through biofilm formation into di- and monosaccharides^2^. In the fluid fraction, fermentation of smaller molecules intensifies, and end-products of fermentation are highly produced by microorganisms and used by the host in metabolic processes^4^. Therefore, the adequate characterization of the ruminal microbiome is essential for developing efficient diagnostic tools and therapeutic interventions to improve animal health and advance the current knowledge on major nutritional issues faced by the dairy and beef industries^5,6^.

Historically, rumen cannula has been the gold-standard method used to investigate the interactions of microbes and feed in these forestomaches that play a pivotal role in ruminants' digestion^3^. Recently, several studies used less invasive methods to characterize high-throughput data in larger populations, which is unprecedented with rumen cannulation^7–9^. However, it remains unclear how these less invasive methods represent microbes and metabolites associated with specific fractions of rumen content (i.e., particulate and fluid) previously reported in rumen-cannulated studies^10^. Amongst the less invasive methods, a collection using an oro-esophageal tube^7^ became a popular technique to obtain rumen contents without the need for major surgery and associated risks and costs with rumen cannulation. The advance in sequencing methods led to studies investigating various aspects of the rumen microbiome and metabolome^11–14^. However, studies comparing the rumen microbiome derived from oro-esophageal and rumen cannula sampling techniques have been controversial, suggesting either consistent^15,16^ or biased results between these two techniques^17,18^. Furthermore, no study assessed the rumen metabolome concurrently with the rumen microbiome for these different sampling techniques to assess the feasibility of the less invasive oro-esophageal approach for multi-omics studies.

Considering the necessity of a large number of animals for proper characterization of cows' genotype and phenotype for rumen multi-omics studies, we propose a study to test the hypothesis that rumen samples collected using oro-esophageal tubing yield a microbiome and metabolome similar to the whole content from rumen cannula, but it is distinct from the cannula fluid and particulate fractions alone. The aims of the study were to characterize the microbiome and metabolome of rumen samples collected using an oro-esophageal tubing and the whole content from the rumen cannula, including the cannula fluid and particulate fractions. Here, we showed that oro-esophageal sampling can be a proxy to screen the 16S rRNA rumen microbiome compared to the rumen cannula technique. Furthermore, results from this study indicate that 16S rRNA sequencing technique for the characterization of the rumen microbiome presents a large within-group variation in both the oro-esophageal tubing and rumen cannula sampling methods, which should be accordingly addressed independently of the chosen sampling method. An under or over-representation of ruminal metabolites and specific ruminal metabolic pathways depending on the sampling method to be used are also discussed below. Overall, this work may help in the design of future rumen microbiome and metabolome studies by addressing pertinent sampling methodological questions discussed in the literature.

## Results

### Rumen pH and upstream denoising analyses

**Figure 1:**
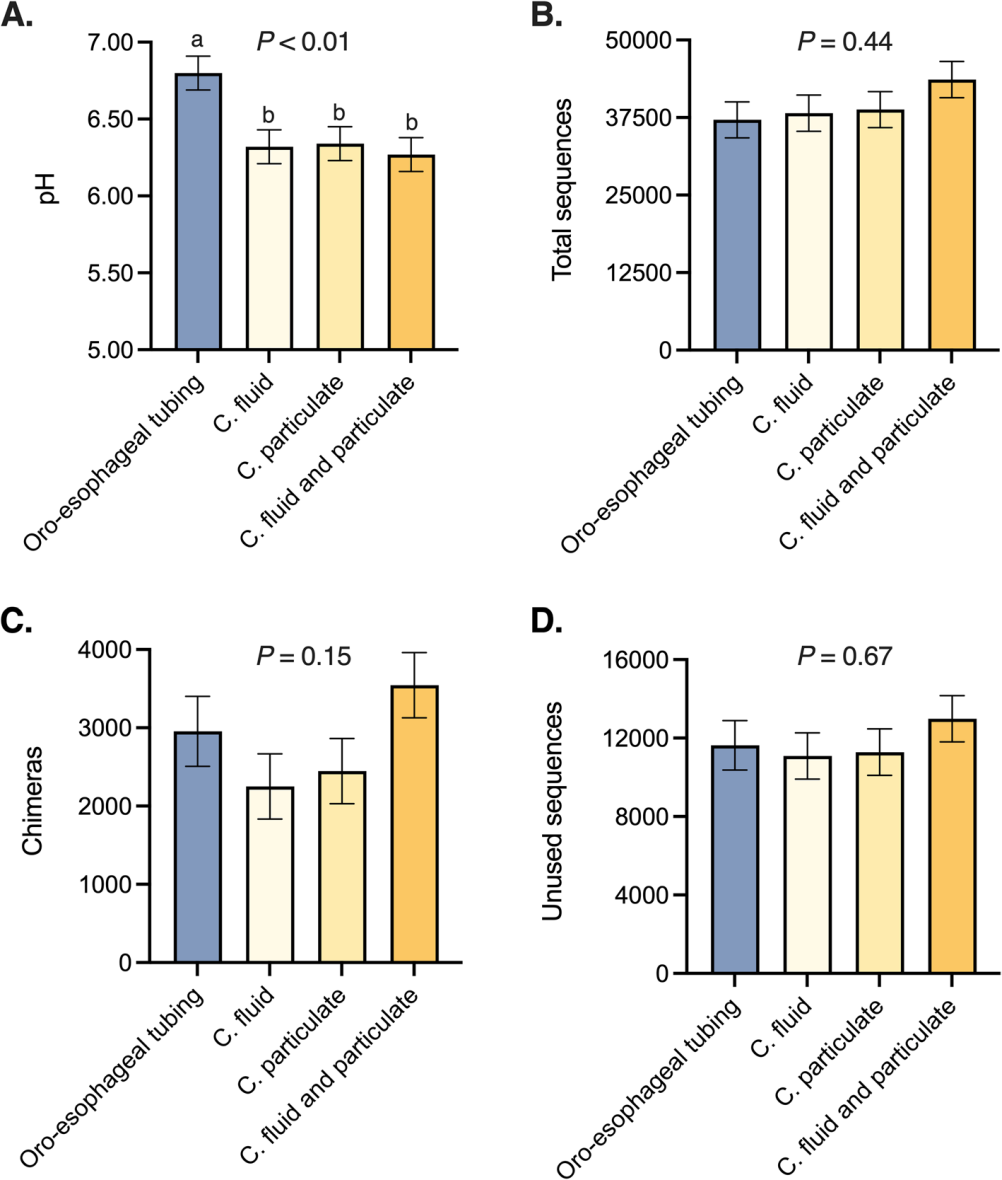
Rumen pH (**A**) and upstream denoising pipeline output metrics displaying total sequences (**B**) number of chimeras (**C**) unused sequences (**D**) amongst oro-esophageal tubing and the respective fractions from rumen cannula. Statistical differences across group means were declared at *P* ≤ 0.05. Different superscripts mean groups differ through the Tukey–Kramer test performed at *P* ≤ 0.05 significance level.

Rumen samples pH ranged from 6.3 to 6.8 amongst different sampling methods, from which rumen content pH collected through an oro-esophageal tube was greater than the pH of ruminal contents collected by rumen cannula (Fig. 1A). However, no significant difference was observed in the quality of total sequencing data analysis amongst sampling methods. Dairy cows in the study had, on average, 39,292 unique ruminal microbial sequences identified with no statistical significance detected amongst sampling techniques (Fig. 1B). There was also no difference in the number of chimeras (Fig. 1C) and the number of unused sequences (Fig. 1D) across sampling techniques, indicating that these methodologies do not differ in the addition of noise to microbiome analysis.

**Figure 2:**
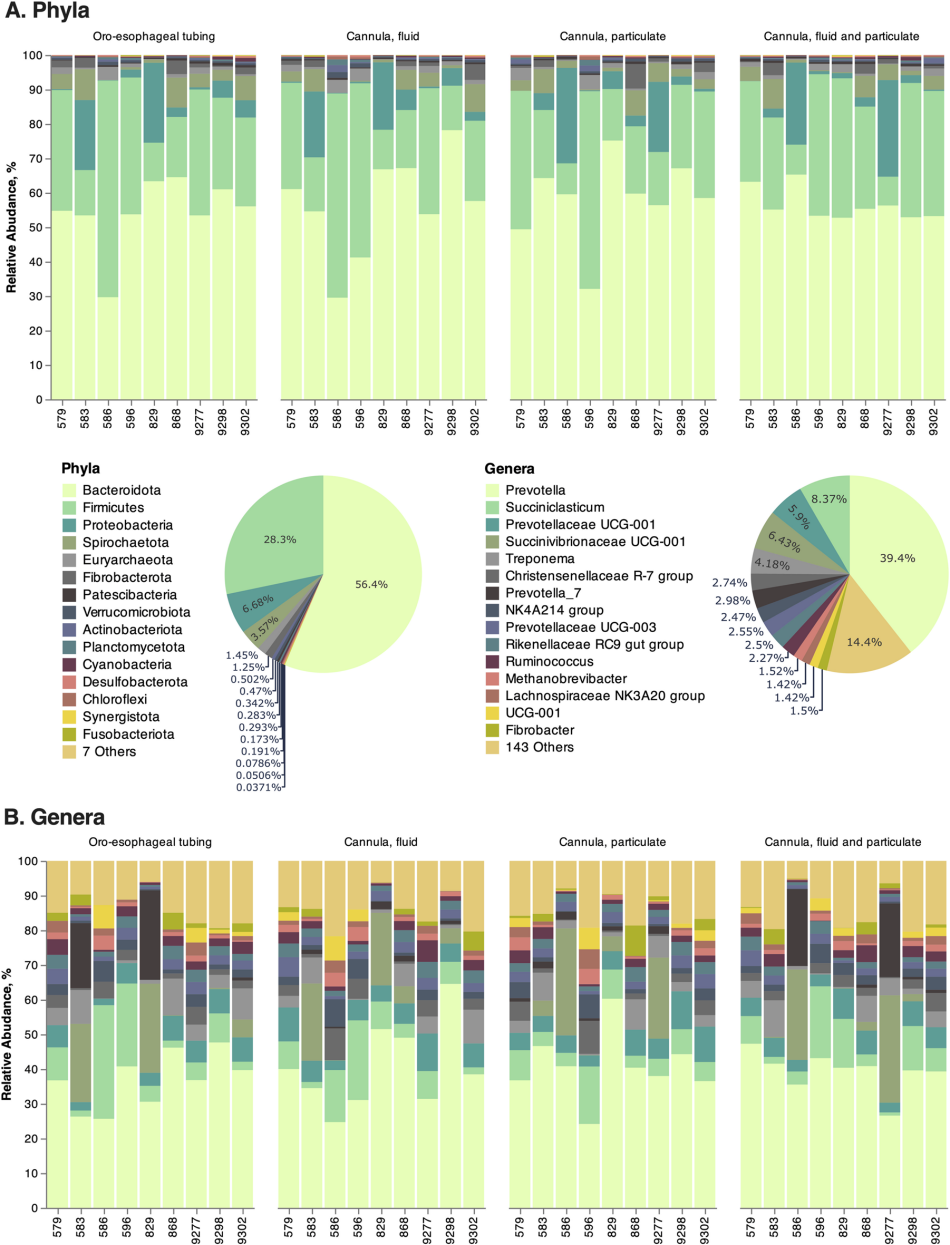
Descriptive analyses of the rumen microbiome composition at the phylum (**A**) and genus (**B**) taxonomy levels in high-producing Holstein cows. Rumen sampling was performed using an oro-esophageal tubing procedure to compare microbiome differences with samples collected from the rumen cannula. The latter was represented as a whole (fluid and particulate) and with the two fractions separated. Data show that the 16S rRNA sequencing technique has large variation independently of the sampling method, suggesting this issue may be mitigated through oro-esophageal sampling that allows a considerable increase in experimental units for a more consistent representation of the overall microbial population.

A total of 7416 ruminal microbial taxa were identified after taxonomy assignment, but no statistical difference was also observed amongst sampling techniques at the phyla and genera levels. The most abundant identified phyla were *Bacteroidetes*, *Firmicutes*, and *Proteobacteria*, while at the genus level *Prevotella*, *Succiniclasticum*, and *Prevotellaceae UCG-001* were the most abundant (Fig. 2).

### Downstream analyses of the rumen microbiome

**Figure 3:**
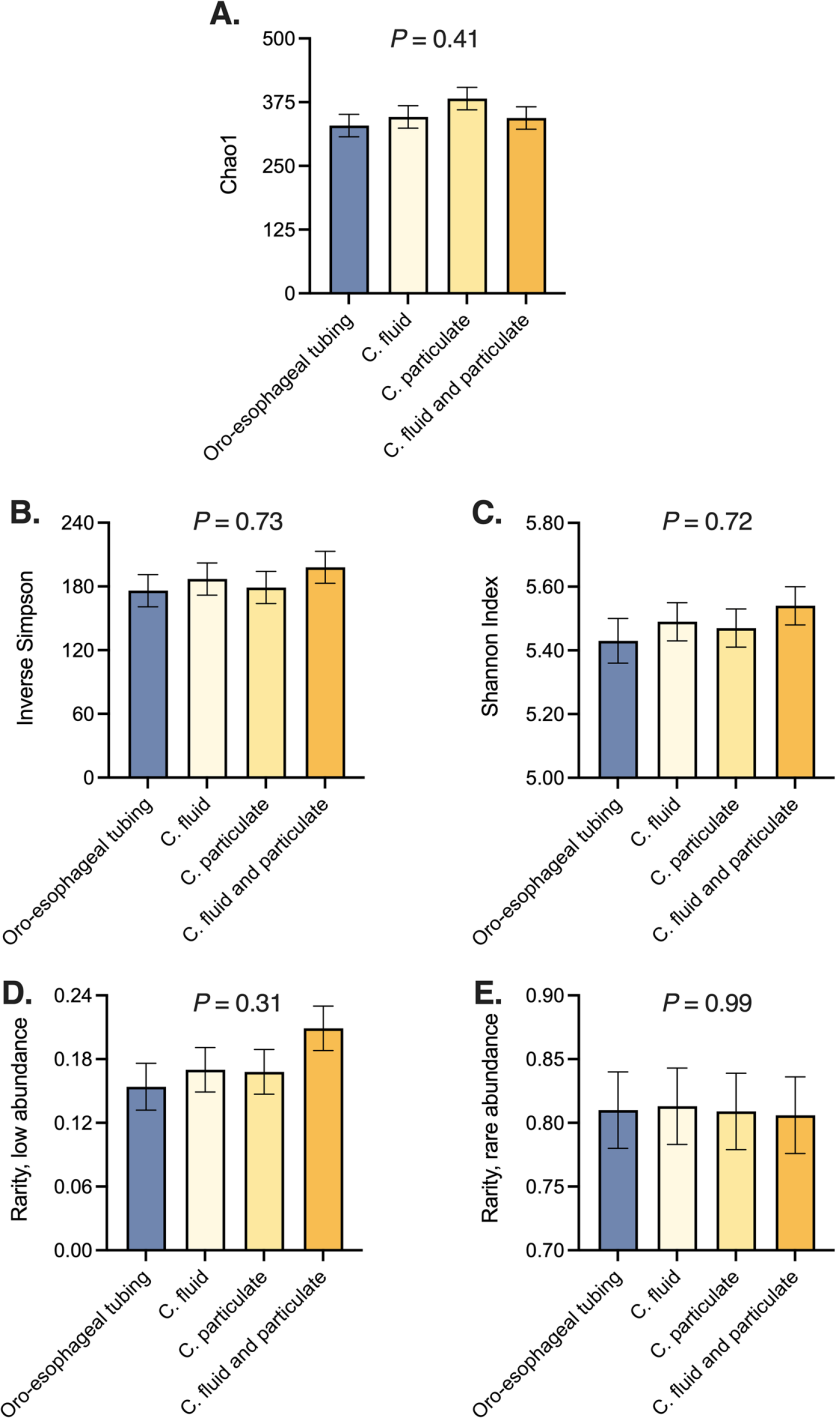
Downstream analyses for alpha-diversity microbiome metrics displaying Chao1 (**A**), Inverse Simpson (**B**), Shannon Index (**C**), rarity index [low (**D**) and rare (**E**) mean relative abundances] microbial taxa amongst oro-esophageal tubing and the respective fractions from rumen cannula. Statistical differences across group means were declared at *P* ≤ 0.05.

**Figure 4:**
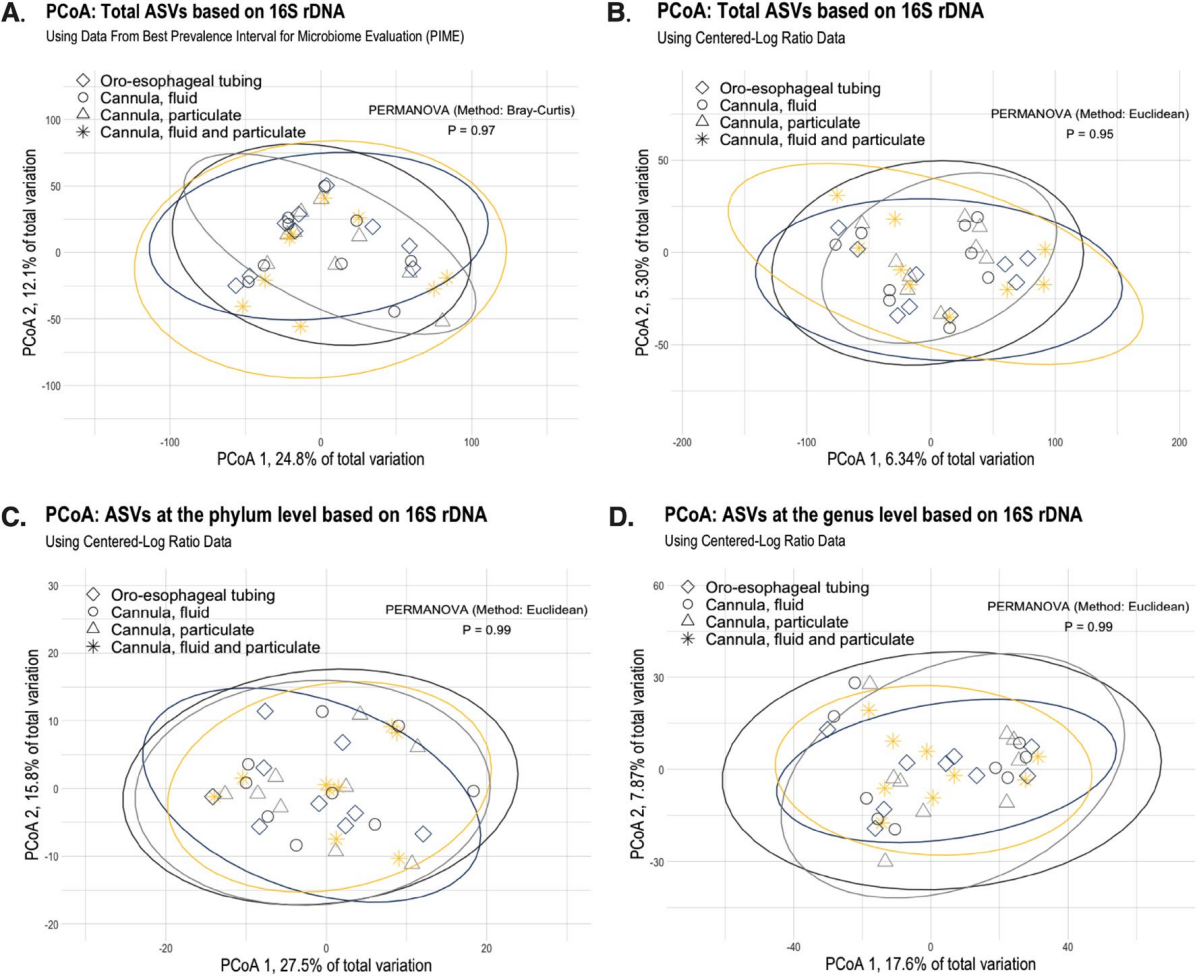
Principal coordinate analysis (PCoA) of the bacterial community composition using: (**A**) prevalence interval for microbiome evaluation (PIME) filtered data to remove noise from taxa not prevalent within sample groups; (**B**) using all microbial taxa (ASV) from centered-log ratio normalization; and the latter (**C**) at the phylum, and (**D**) genus taxonomy levels. Statistical differences for permutational multivariate analysis of variance (PERMANOVA) were declared at *P* ≤ 0.05.

Alpha diversity indexes are described in Fig. 3A–E. Chao1, Shannon, Inverse Simpson, and rarity (low and rare abundances) indexes did not differ amongst oro-esophageal tubing and rumen cannula techniques. For beta-diversity, no differences were detected in the rumen microbiome composition among the different sampling methods, as shown by principal coordinate analyses (Fig. 4A–D). Permutational multivariate analysis of variance and LEfSe also revealed no significant microbial taxa difference between oro-esophageal tubing and rumen cannula techniques. Furthermore, no difference was observed amongst these techniques for the 22 identified phyla. No difference in genera mean relative abundance was detected amongst sampling methods as well, nor were there differences amongst rumen source in the discriminant analysis.

### Rumen metabolome

**Figure 5:**
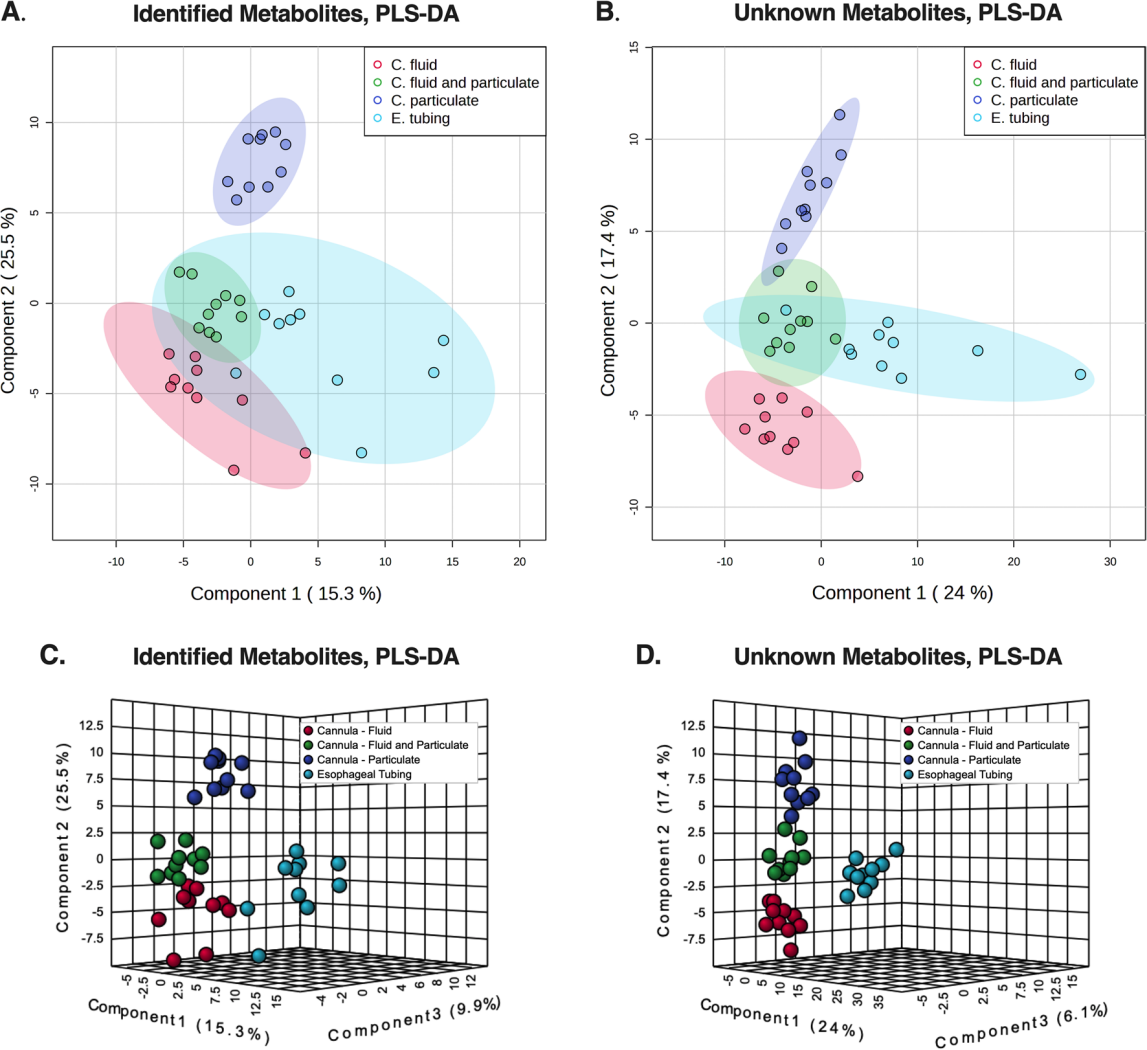
Partial least square-discriminant analysis (PLS-DA) of ruminal metabolites from ruminal samples collected through an oro-esophageal tubing and the respective fractions from the rumen cannula. A 2-D representation of differences in known (**A**) and unknown (**B**) metabolome composition is displayed for the illustration of how closely related the oro-esophageal tubing metabolome samples are to the respective fractions from the rumen cannula. A 3-D representation of known (**C**) and unknown (**D**) metabolome composition is displayed to demonstrate that there are still differences between the oro-esophageal tubing procedure and rumen cannula metabolome samples that need to be considered in future studies.

A total of 185 knowns and 236 unknown primary metabolites were identified in the rumen content. Partial least squares discriminant analysis (PLS-DA) was performed to evaluate ion abundance metabolite differences among rumen sampling methods (Fig. 5). Overall, PLS-DA with known metabolites indicates the metabolome composition and ion abundance of samples collected through the oro-esophageal tubing procedure is more closely related to the whole content from rumen cannula (Fig. 5A), despite greater dispersion and some metabolite differences as shown by the third principal component in Fig. 5C. Within the rumen cannula technique, PLS-DA with known metabolites indicate the metabolome composition of samples containing only the fluid fraction is considerably different from those containing only the particulate fraction. The PLS-DA for unknown metabolites illustrated a similar pattern of distinction as in the composition of known metabolites. A moderate overlap of oro-esophageal samples with the whole content from the rumen cannula was also observed in major principal coordinates (Fig. 5B); however, sampling methods yielded more homogeneous metabolite composition for unknown than known metabolites (Fig. 5C,D). Nonetheless, the oro-esophageal tubing procedure had considerably greater data dispersion than all samples from the rumen cannula, indicating that the former may require more experimental units to have a representative overview of the population compared to the latter.

**Figure 6:**
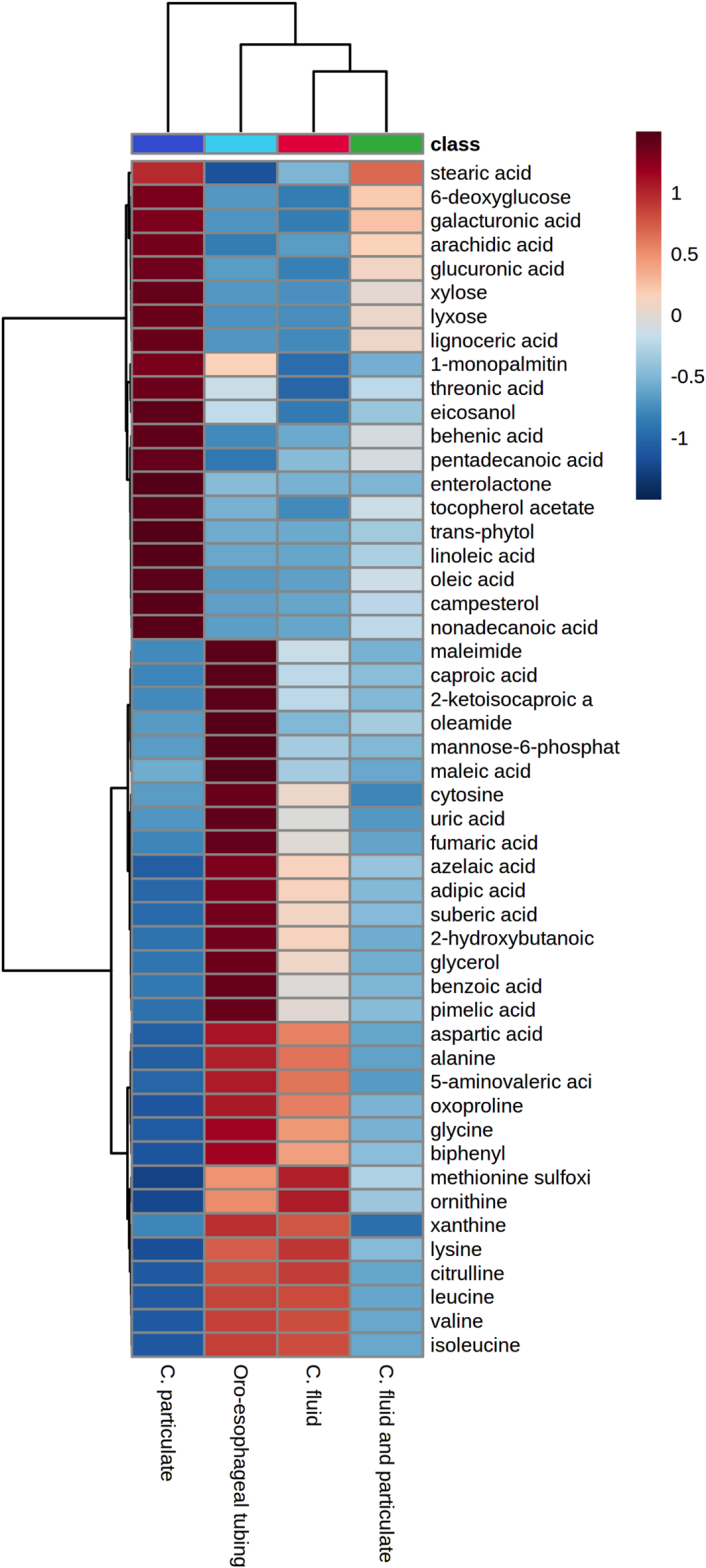
Hierarchical clustering heatmap showing top 50 metabolites detected through analysis of variance to differ in rumen sampling methods. Color differences indicate the Pearson correlation of metabolite ion abundances and sampling method. Wards clustering method was used to assess similarity among sampling methods and is displayed at the top portion of the heatmap. Statistical differences were declared at *P* ≤ 0.05. Heatmap was produced on Metaboanalyst 5.0 (https://www.metaboanalyst.ca/).

**Figure 7:**
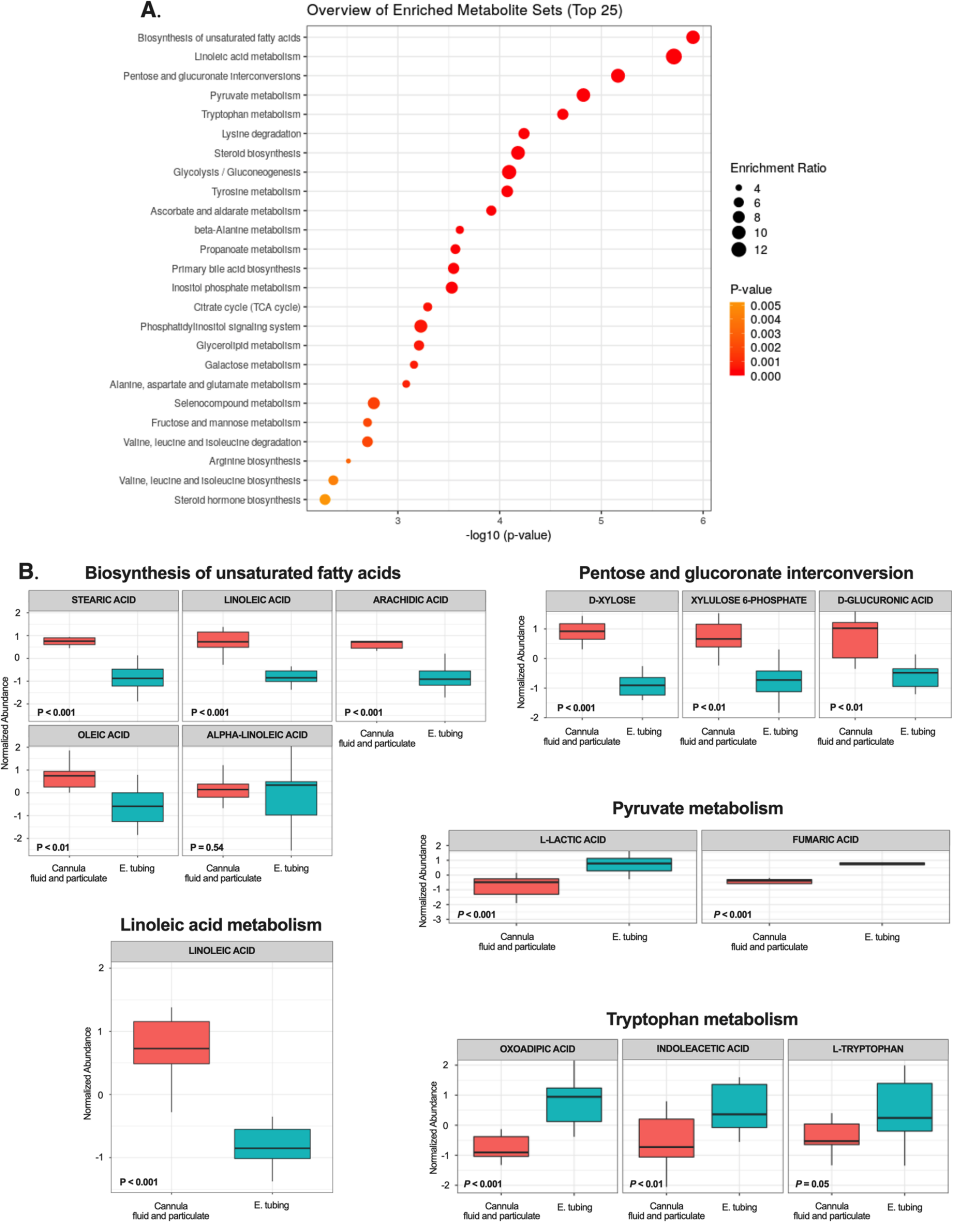
Enrichment pathway analysis was performed in Metaboanalyst 5.0 using the KEGG pathway database^45–47^. The figure displays the top 25 most enriched pathways (**A**) that differed between the oro-esophageal tubing procedure and a complete sample from the rumen cannula (fluid and particulate together). (**B**) graphical visualization of metabolite differences between the two sampling techniques within some of the top 25 most enriched pathways. Statistical differences were declared at *P* ≤ 0.05.

Hierarchical Wards clustering of sampling methods based on major known metabolites detected in rumen samples as well as the correlation of these metabolites with sampling methods, are shown in Fig. 6. Based on Ward's hierarchical clustering, the ion abundance of major known metabolites from oro-esophageal tubing technique samples were also similar to samples from rumen cannula. However, correlation analyses showed the ion abundance of these major metabolites (top 50 from T-test and ANOVA; *P* < 0.05) from oro-esophageal tubing technique samples were more similar to those of the fluid fraction from rumen cannula, which would be the traditional strained rumen fluid widely used in nutritional studies. Lastly, enrichment pathway analysis based on identified metabolites allowed the understanding of whether any of the sampling methods would affect or not nutritional studies in ruminants. The 25 major pathways that can potentially be affected between the oro-esophageal tubing technique and rumen cannula are shown in Fig. 7A, and some of those are reported in more detail in Fig. 7B. One of the major pathways that could potentially be affected was the biosynthesis of unsaturated fatty acids, which would represent chances of potentially having different yields of these unsaturated fatty acids from the rumen depending on the chosen sampling methods. The ion abundances of stearic, linoleic, arachidic, and oleic acids were greater in rumen cannula samples than in the oro-esophageal tubing ones (Fig. 7B). The pathway associated with linoleic acid metabolism was the second most enriched pathway followed by the pentose and glucuronate interconversions (Fig. 7B), where the ion abundances of d-xylose, xylulose 5-phosphate, and d-glucuronic acid were greater in the rumen cannula samples than oro-esophageal tubing ones. Following the pathways of pyruvate and tryptophan metabolism with the ion abundances of oxoadipic acid, indoleacetic acid, l-tryptophan metabolites greater in oro-esophageal samples than in the rumen cannula.

## Discussion

The current study sheds light on the impact of different sampling methods to characterize the rumen microbiome and metabolome concurrently in dairy cows. Here, the microbiome findings suggest that studies designed to use oro-esophageal tubing to collect rumen samples for microbiome evaluation can yield similar results compared to samples collected through the rumen cannula. Despite the similarities for the rumen microbiome, these data also suggest that the overall rumen metabolome of the oro-esophageal procedure can be represented by the combined fluid-particulate fractions collected from the rumen cannula, but that the metabolome composition of the independent fractions from the cannula is distinct. These findings highlight that methodologies and which rumen fractions to be used are important considerations when studying specific metabolites. Nonetheless, the microbiome and metabolome of oro-esophageal and rumen cannula fluid-particulate combined samples were generally similar, highlighting once again the interchangeable similarity between both methods when evaluating ruminal microbial and metabolic changes.

As expected and previously demonstrated^19^, the pH of rumen samples was greater in the oro-esophageal tubing than in the contents from the rumen cannula. The findings of the current study confirm that samples collected with an oro-esophageal tubing may, in fact, present a higher pH due to saliva contamination. Still, despite this difference, the overall microbiome for the oro-esophageal and rumen cannula fluid-particulate combined samples did not diverge. Therefore, if the goal of researchers is to simply characterize the rumen microbiome changes using 16S rRNA sequencing in a single time in relation to feeding, the current findings suggest that the variation in pH has a negligible impact on the characterization of the rumen microbiome present in fluid and particulate fractions of the rumen.

The effect of rumen fractions and the methodologies were not significant for most bacterial communities. Even the richness of particulate and fluid-particulate samples containing degraded fiber when compared to fluid did not differ from rumen contents and between sampling methods as described before^16,20^. These results are in agreement with other studies that compared these methodologies to collect rumen samples at different hours after feeding^20^, at different sites^20^, and in pre-weaned calves^21^. However, Deusch et al.^22^ found a significant change (*P* < 0.05) in bacterial community arrangement over different rumen fractions and diets, which is probably more likely associated with the different diets than sites.

The most prevalent phyla were consistently *Bacteroidetes* followed by *Firmicutes* in oro-esophageal and rumen contents collected using the cannula^16,22^. In a study comparing the rumen microbiome from 32 different ruminant species in 35 different countries worldwide^23^, these two phyla were the most abundant and part of a core microbiome considering variations in diet and host. Specifically evaluating sampling methodologies, De Assis and collaborators (2020)^20^ found an increase in the relative abundance of *Firmicutes* and a decrease of *Bacteroidetes* in samples from rumen cannula when compared to stomach tubing over time. The differences in the bacterial communities from De Assis and collaborators^20^ were reported to be associated with the time of collection (no differences up to 4 h) post-feeding between oro-esophageal tubing and rumen cannula. In the current study, the lack of difference was noted at 5 h in a single time point collection likely due to the lack of repeated opening of the rumen cannula and introduction of oxygen and disturbances to the rumen microbiome. Thus, precautions to avoid repeated interaction with rumen contents and exposure to oxygen may mitigate potential causes of differences between the two techniques. Also, in the current study, the *Bacteroidetes* to *Firmicutes* ratio was not significantly different (*P* = 0.97) among sampling techniques, indicating such variation may not happen in a more controlled setting. At the genus level, *Prevotella* was the most abundant genus found in the rumen samples of the current study, which has been widely reported in other rumen studies as well^8,9,16^, including rumen microbiome studies with a large number of samples analyzed^23^. *Prevotella* is one of the major microbes responsible for the degradation of starch and protein and plays an essential role in volatile fatty acids biosynthesis. This genus is also associated with cows with high milk yield and milk protein content which were the characteristics of the cows used in our study^14^.

Another difference observed by De Assis and collaborators^20^ was regarding the variation in beta-diversity in samples from the oro-esophageal technique and those collected using rumen cannula, showing a larger variation in the former. Differences in group microbiome variation were not observed in the current study, but the metabolome had a similar larger variation that is described later in this discussion. Henderson et al.^24^, comparing different methodologies for the extraction and sequencing of rumen bacteria and archaea communities, show that there is a variation depending on the protocol to be used but that this variation is not large enough to be present in dimension reduction analysis such as the PCoA reported here. For the current study, we went even further and tested different methods to detect microbiome differences (PIME, Phylum, and Genus PCoAs) and did not find differences. In both cases, even considering such variation in previous microbiome studies, the group centroid of populations is similar and often overlaps in these analyses, meaning differences between the oro-esophageal tubing technique and the whole sample from rumen cannula considering a reasonable sample size may not be as large as previously reported. Other factors that could potentially change ruminal parameters and need careful consideration when using the oro-esophageal technique are the depth of the inserting tube, and the tube length used to collect the rumen content^24,25^. The tube used in the current study had openings large enough to pass particulate fractions that could account for microbial populations attached to feedstuff, which overall may have contributed to such small differences in microbial populations from this study. In sum, our descriptive analysis shows that variation in 16S rRNA rumen microbiome studies is likely introduced by the 16S rRNA technique itself, as despite similar variation between groups, microbiome populations were highly variable even within rumen cannula groups. This is a consequence of compositional datasets, which may have even more variation introduced depending on how data is analyzed^26^. Thus, the report of descriptive data within groups is highly advisable to enhance the discussion of not only the proper sampling technique to be used but to avoid reliability issues of any outcomes from microbiome studies. In this scenario, a larger number of experimental units in rumen microbiome studies may be one of the only ways to represent the overall targeted population better, and this approach is likely more feasible only through the oro-esophageal tubing procedure.

Regarding metabolite dispersion across different sampling methods, the current study revealed that oro-esophageal tubing procedure and rumen cannula have similar compositions, with distinctions regarding specific metabolites and some pathways, as shown for the microbiome in a previous study^20^. Therefore, even though variation in sample composition exists, the centroids representing the overall group mean from the oro-esophageal tubing procedure and rumen cannula from major metabolite variation were similar, suggesting that larger sample size studies, which is a reason and potential advantage of using the oro-esophageal technique, may help reduce the misrepresentation of populations. For specific metabolites, large differences were observed mainly between the fluid and particulate fractions of the rumen content collected through the rumen cannula, possibly due to the nature of fractions and nutrients that generate their respective end-products of fermentation. However, when considering samples that contain similar physical composition, such as the oro-esophageal and combined fluid-particulate from the rumen cannula, the difference is mostly associated with an overall contribution of some major metabolite variations, which changed some metabolic pathways as reported here.

Sampling through the rumen cannula has been traditionally used because of the direct assessment of the rumen content and the expectancy of a more reliable representation of the native composition of the rumen. In the case of the oro-esophageal tubing sample that is not filtered, the opening in the tip of the collection tube also allows the collection of a representative fraction of the rumen. This might explain why the rumen metabolite composition from the oro-esophageal technique was similar to the fluid-particulate samples collected by rumen cannula. However, because the sample is not taken as a mixed one from all sites of the rumen but at random, a slight variation was introduced, and more samples may be necessary to characterize metabolome phenotypes more accurately. Thus, due to the close relationship between ruminal metabolites with different pathways and even their direct presence in different ones^27^, the current study suggests rumen metabolome studies should be carefully designed, and an adequate number of experimental units can be a factor to be considered to avoid such problems.

An example is unsaturated fatty acids, which are synthesized by aerobic and anaerobic mechanisms depending on the organism^28^. Not only the oxygen but the environment, temperature, and nutrition can modify the composition of the lipid molecule^29^. These factors can explain the greater ion abundance of these metabolites (stearic, linoleic, arachidic, oleic, and alpha-linolenic acids) in combined fluid-particulate samples than in oro-esophageal samples. Changes in the rumen environment due to the oxygen circulation through the cannula for this specific pathway may alter lipid metabolism pathways. In this case, the oro-esophageal technique may be an advantageous approach for characterizing specific lipids. The difference in unsaturated fatty acids exemplifies how these changes in metabolome composition are less likely to follow the same pattern as those found in the microbiome. There are also metabolites derived from other microorganisms, different plant materials, or even the host^11^, which could potentially change the study's outcome, but the contribution of these factors was beyond the scope of the current study.

In conclusion, the current study indicates that despite having greater rumen content pH, the oro-esophageal procedure did not present major microbiome or metabolome composition differences when compared to the whole content from the rumen cannula. Furthermore, the 16S rRNA sequencing technique for the characterization of the rumen microbiome presented large variation in both the oro-esophageal tubing and rumen cannula sampling methods, which should be accordingly addressed independently of the chosen sampling method. For the rumen metabolome, small variations in some rumen metabolites may potentially change specific metabolic pathway outcomes in the rumen. Thus, studies looking at specific ruminal pathways associated with the rumen microbiome should carefully consider the sampling method to be used in order to draw adequate conclusions regarding metabolite abundances and metabolic pathway changes in the rumen.

## Methods

All experimental procedures were conducted at the University of Illinois at Urbana-Champaign and followed protocols approved by the Institutional Animal Care and Use Committee (IACUC) at the University of Illinois at Urbana-Champaign under protocol number 17172. All ARRIVE, and IACUC guidelines and regulations were followed during the entire duration of the study. Ten cannulated multiparous high-producing Holstein cows in mid-lactation averaging 688 ± 78 kg BW were enrolled in the study. All cows were housed in a tie-stall system with sand bedding, fed twice a day ad libitum, and had free water access at all times. A period of 14 days was used to adapt the cows to the diet before sampling. Diets were formulated using AMTS.Cattle.Pro version 4.7 (2017, AMTS, LLC, Groton, NY) to meet or exceed recommendations for cows producing 41 kg of milk/d with a target of 3.8% milk fat and 3.2% milk protein and a predicted DMI of 25 kg/d. The diet fed consisted of corn silage, alfalfa hay, soybean meal, dry ground corn grain, canola meal, corn gluten feed, soy hulls, dried molasses, bypass fat, premixed vitamins and minerals (Vitamin and mineral mix was formulated to contain 13.50% Ca, 0.001% P, 3.92% salt, 10.90% Na, 6.68% Cl, 2.33% Mg, 8.27% K, 0.14% S, 1.77 mg/kg Co, 126.98 mg/kg Cu, 32.86 mg/kg I, 602.01 mg/kg Fe, 980.85 mg/kg Mn, 7.47 mg/kg Se, 3.15 mg/kg organic Se, 888.79 mg/kg Zn, 108.86 kIU/kg Vitamin A, 21.77 kIU/kg vitamin D3, 410.51 IU/kg vitamin E, 2.48 mg/kg choline, 18.21 mg/kg biotin, 0.16 mg/kg Niacin, 0.004 mg/kg thiamine.), rumen-protected lysine and methionine, and urea 46%.

### Sampling procedure

Rumen samples were collected 5–6 h after morning feeding, and samples (oro-esophageal content, fluid, particulate, and combined fluid-particulate cannula) were collected from each of the ten cows enrolled in the study totalizing 40 samples. Briefly, an oro-esophageal sampling device was used to collect rumen content samples^7^. A vacuum pump equipped with a glass container was connected to a tube of approximately 200 cm in length and 2.5 cm in diameter before being used. The tube was inserted orally in the cows until it could reach the rumen. Rumen content was collected through building vacuum pressure in the tube. The first two samples were discarded to avoid contamination of rumen contents with esophageal components, such as saliva and mucus. Then, approximately 500 mL of rumen content was collected, and 15 mL of the content was immediately placed in sterile conical tubes and frozen in liquid nitrogen until further analysis. Before the sample collection from each cow, the whole oro-esophageal tube and collecting container were thoroughly cleaned with current water, followed by immersion in chlorhexidine solution to minimize cross-contamination. Samples from the rumen cannula representing the combined cranial, caudal, dorsal, and ventral regions of the rumen equally sampled (approximately 125 mL) were collected according to their respective fractions: fluid and particulate fractions separately and a homogeneous sample containing both fractions as a proxy to the overall composition of rumen contents. In brief, a homogenous fraction was collected and squeezed through two layers of cheesecloth saving the fluid (15 mL) and particulate (50 mL) contents in separated containers. Then, a homogenous fraction was collected through the rumen containing 50 mL of the combined fluid and particulate contents. During all collections, ruminal pH was measured using a portable pH meter immediately after sampling. Samples were immediately frozen in liquid nitrogen and transported to the laboratory in Urbana, IL, where they were kept at − 80 °C freezer until further analyses.

### DNA extraction, library preparation, and sequencing

Bacterial DNA was extracted similarly to Lima et al.^8^. Briefly, rumen samples were thawed at 4 °C and later centrifuged for 10 min at 16,000 RCF in a DNase-free microcentrifuge tube. The supernatant was discarded, and the pellet was resuspended in nuclease-free water. A QIAamp PowerFecal DNA Extraction Kit (Qiagen) was used for genomic DNA isolation. Except for the addition of 400 mg of lysozyme during bacterial resuspension and the following incubation of 12 h at 56 °C to maximize bacterial DNA extraction, all other manufacturer’s instructions were followed for genomic venal isolation. A NanoDrop ND-1000 spectrophotometer (NanoDrop Technologies, Rockland, DE, USA) was used later at wavelengths 230, 260, and 280 nm for DNA concentration and purity measurements.

Library preparation and sequencing were performed similarly to those described by Kozich et al.^30^. Amplification was performed through polymerase chain reaction (PCR) in a Bio-Rad C1000 TouchTM Thermal Cycler (BIO-RAD, Hercules, CA, USA). The V4 region of the 16S rDNA gene was amplified using the Earth Microbiome Project barcoded (forward: GTGYCAGCMGCCGCGGTAA and reverse: GGACTACNVGGGTWTCTAAT) bacterial primers through an initial 95 °C denaturation for 5 min, followed by 30 cycles of 30 s at 95 °C, 30 s at 55 °C, 1 min at 72 °C, and 5 min for final elongation at 72 °C. Primers and small DNA fragments were removed using a 1% low melting agarose gel extraction kit (National Diagnostics, Atlanta, GA, USA). Purification and normalization of amplicons were performed using a SequalPrep plate kit (Invitrogen, USA), and the DNA concentration was measured with a Qubit Fluorometer. Adapters were added to the amplicons, and a DNA library was prepared by equally pooling them together; qualitative real-time PCR was used for a quality check. A total of 40 samples were sequenced using an Illumina MiSeq 2500 platform.

### Metabolomics data acquisition and processing

Ruminal metabolites were extracted following the procedure of Fiehn et al.^31^ and analyzed in a gas chromatography time-of-flight mass spectrometer (GC-TOF)^31^. The retention index and the complete mass spectrum were encoded as a string. All thresholds reflect settings for ChromaTOF v. 2.32. Quantification was reported as peak height using the unique ion as the default unless a different quantification ion was manually set in the BinBase administration software BinView. We detected 185 known metabolites from a total of 421 untargeted primary metabolites found in our analysis. A column of 30 m length by 0.25 mm internal diameter with 0.25 μm film made of 95% dimethyl/5diphenyl polysiloxanesne was used in a Restek corporation Rtx-5Sil MS. The gas helium (99.99% purity) was used a carrier for the analysis, and the column temperature was set between 50 and 330 °C at flow-rate of 1 mL min^−1^. The oven temperature was set to 50 °C for 1 min, then ramped at 20 °C min^−1^ to 330 °C, and held constant for 5 min. Finally, the injection temperature was set to 50 °C and ramped to 250 °C by increments of 12 °C^−1^. The retention of primary metabolites (amino acids, hydroxyl acids, carbohydrates, sugar acids, sterols, aromatics, nucleosides, amines, and miscellaneous compounds) were evaluated.

### Bioinformatics and statistical analyses

The first step in our bioinformatic analyses was the preparation of our metadata. For that, different sources of rumen samples were the groups for comparison. Downstream analysis was performed by testing differences between bacterial communities of each group created in the metadata. Upstream and downstream analyses of the sequenced amplicons were mostly performed in R Studio 2021.09.1. Sequences were denoised using the *dada2* pipeline^32^, in which demultiplexed fastq files were inspected, filtered, and trimmed based on their quality scores and error rates. Chimeras were removed, and an ASV table was created. Taxonomy was assigned using the 16S rRNA SILVA v138 database^33^ with the *phyloseq* package^34^. Total taxa were then split into taxonomy levels, and the relative abundance of the ASVs within each taxonomy level was calculated using the *phyloseq* package. One microbiome oro-esophageal tubing sample had almost null counts, likely due to library preparation, and was discarded from the remaining analyses. Alpha-diversity indexes [(total sequences, chimeras, unused sequences, Shannon, Chao 1, Inverse Simpson, and Rarity (low and rare abundant taxa)] were calculated using the *microbiome* and *vegan* packages^35,36^.

Data were normalized using Center-Log Ratio (CLR) transformation^26,37–39^ for the generation of principal coordinate analysis (PCoA) for graphical visualization of beta-diversity differences. Prevalence interval for microbiome evaluation [PIME; *pime* package^40^] was also tested to better select statistically and biologically relevant taxa for beta-diversity analysis. This latter decontamination pipeline allows the filtering of noise within each group by using random forest classification. An error rate indicates the ideal prevalence at which most of the 16S rRNA sequences are kept in the dataset, but the removal of some microbial taxa not consistent within a group contributes to a decrease in noise for further analysis. Based on the indicated appropriate prevalence interval calculated for the tested groups (prevalence of 25% within a group), taxa that were not shared within the same group were removed for a better visualization of the differences among bacterial communities.

Four PCoA plots were constructed using the following: (A) PIME filtered microbial taxa, (B) all microbial taxa (ASV) after CLR normalization, and the latter at (C) the phylum and (D) genus levels. Graphs were generated using the *ggplot2*, *dplyr*, *hrbrthemes*, *viridis*, *ggsci*, and *RColorBrewer* packages. Permutational multivariate analyses of variance [PERMANOVA;^41^] were performed to test the bacterial community’s dispersion differences with the respective datasets from PCoA, and statistical differences were considered at* P* ≤ 0.05. Linear discriminant analysis of effect size [LEfSe;^42^] was used to evaluate taxa differences between each sampling procedure. The LEfSe algorithm is based on three statistical tests (Kruskal–Wallis and Wilcoxon sum-rank tests, and linear discriminant analysis) to declare taxa differences in bacterial communities. However, no difference was observed in the LEfSe analysis and thus, no results were reported in this study.

Metabolomic analyses were performed using Metaboanalyst 5.0^43,44^. In brief, partial-least square discriminant analysis (PLS-DA), hierarchical clustering, and enrichment pathway analyses were performed to understand metabolite differences between sampling techniques. The KEGG metabolites library was used^45–47^, and the top 5 enriched pathways were considered for direct comparisons between the oro-esophageal procedure (newer method of rumen sampling) and the rumen cannula technique (the traditional gold standard for rumen sampling).

Lastly, a model containing the fixed effect of the sampling procedure and the random effect of the cow was fitted in SAS 9.4 for pH, all alpha-diversity variables, and the relative abundance of bacterial taxa. Statistical analyses were considered significant when *P* ≤ 0.05. When a statistical difference was observed, Tukey–Kramer test was used to compare group means, and the same *P*-value threshold was used to define between-group differences.

## Data Availability

Metabolomic data was submitted to Metabolomics Workbench, and amplicon sequences were deposited in the Sequence Read Archive (SRA) of the National Center for Biotechnology Information (NBCI) under access number PRJNA784126.
